# Rhizosphere protists are key determinants of plant health

**DOI:** 10.1186/s40168-020-00799-9

**Published:** 2020-03-03

**Authors:** Wu Xiong, Yuqi Song, Keming Yang, Yian Gu, Zhong Wei, George A. Kowalchuk, Yangchun Xu, Alexandre Jousset, Qirong Shen, Stefan Geisen

**Affiliations:** 1 Jiangsu Provincial Key Lab of Solid Organic Waste Utilization, Key Lab of Plant Immunity, Jiangsu Collaborative Innovation Center of Solid Organic Wastes, Educational Ministry Engineering Center of Resource-saving fertilizers, Nanjing Agricultural University, Nanjing, 210095 Jiangsu People’s Republic of China; 2 Ecology and Biodiversity Group, Department of Biology, Institute of Environmental Biology, Utrecht University, Padualaan 8, 3584 CH Utrecht, The Netherlands; 3 Department of Terrestrial Ecology, Netherlands Institute for Ecology (NIOO-KNAW), 6708 PB Wageningen, The Netherlands; 4 Laboratory of Nematology, Wageningen University & Research, 6700 ES Wageningen, The Netherlands

**Keywords:** Rhizosphere, Pathogen of *Ralstonia solanacearum*, Protists, Predator-prey interactions, Secondary metabolite genes, Plant health

## Abstract

**Background:**

Plant health is intimately influenced by the rhizosphere microbiome, a complex assembly of organisms that changes markedly across plant growth. However, most rhizosphere microbiome research has focused on fractions of this microbiome, particularly bacteria and fungi. It remains unknown how other microbial components, especially key microbiome predators—protists—are linked to plant health. Here, we investigated the holistic rhizosphere microbiome including bacteria, microbial eukaryotes (fungi and protists), as well as functional microbial metabolism genes. We investigated these communities and functional genes throughout the growth of tomato plants that either developed disease symptoms or remained healthy under field conditions.

**Results:**

We found that pathogen dynamics across plant growth is best predicted by protists. More specifically, communities of microbial-feeding phagotrophic protists differed between later healthy and diseased plants at plant establishment. The relative abundance of these phagotrophs negatively correlated with pathogen abundance across plant growth, suggesting that predator-prey interactions influence pathogen performance. Furthermore, phagotrophic protists likely shifted bacterial functioning by enhancing pathogen-suppressing secondary metabolite genes involved in mitigating pathogen success.

**Conclusions:**

We illustrate the importance of protists as top-down controllers of microbiome functioning linked to plant health. We propose that a holistic microbiome perspective, including bacteria and protists, provides the optimal next step in predicting plant performance.

**Video Abstract**

## Background

Plant pathogens can colonize the rhizosphere and have a severe influence on plant health [1, 2]. However, pathogen success and plant health are ultimately controlled by other biota, particularly the rhizosphere microbiome [3, 4]. The plant rhizosphere microbiome is a complex assembly of diverse microorganisms, including bacteria, fungi, and protists that together influence plant health [5–8]. Despite the fact that the microbiome consists of diverse groups, most research aiming to understand the role of the microbiome in plant health or disease suppression has focused on bacteria [9–11] and fungi [12, 13]. A whole-microbiome view to decipher the main microbial determinants and their potential interactions that determine plant performance is currently missing [14]. As such, a more complete microbiome analysis is needed to identify the microbial groups and potential interactions that help predicting plant health.

In particular, protists that steer the taxonomic and functional composition of the rhizosphere microbiome through trophic predator-prey interactions have so far rarely been included in microbiome analyses linked to plant performance [8]. Protists, especially microbial-feeding phagotrophs [15, 16], have various functions within the rhizosphere [6, 17, 18]. For instance, some of these phagotrophs can directly prey on plant pathogens [19]. Studies using model protists have shown that protists control microbiome diversity and structure leading to plant growth promotion [17, 18, 20]. These changes are at least partly explained by the fact that protists feed selectively on microbial prey taxa, which differs between protistan species [21, 22]. Through this selective predation, protists can, for instance, increase those bacteria that produce pathogen-suppressive secondary metabolites [23, 24]. Yet, all these studies investigating potential links of protists with plant performance were carried out under artificial laboratory or greenhouse conditions focusing on one or few protistan species. As such, we have yet to identify the links between a complex diversity of protists, the microbiome and plant performance, especially in agricultural systems under field conditions.

Protists and their interactions with other microorganisms are also subject to change throughout plant growth [14, 25]. Yet, the composition of the microbiome is often investigated only once during plant growth, usually at the time of plant maturity or after disease has already developed. Such approaches make it difficult to disentangle causality between plant health and potentially underlying characteristics in microbial communities, especially for diseased plants that host high amounts of pathogens. Recently, it was shown that bacterial communities at plant establishment can predict plant health at maturity [26]. Yet, other microbial groups might be even better indicators to predict plant health, as for instance, protist communities were shown to respond more strongly to environmental inputs and vary more in their composition between seasons than bacteria and fungi [27].

To investigate potential key microbiome groups that might predict plant health, we here used a rhizobox system in an agricultural system under field conditions, in which we grew tomato plants. Soils were naturally infested with pathogenic *Ralstonia solanacearum* bacteria, one of the most devastating and globally distributed soil-borne plant pathogens that can infect a range of important crops [28, 29]. In the rhizosphere of plants that either later developed disease symptoms or remained healthy, we temporally investigated the microbiome composition, including bacteria, fungi, and protists, as well as potential microbial functions using metagenomics. We tested the hypothesis that protists rather than other microbial communities in the rhizosphere microbiome best predict pathogen dynamics and plant health.

## Results and discussion

**Fig. 1:**
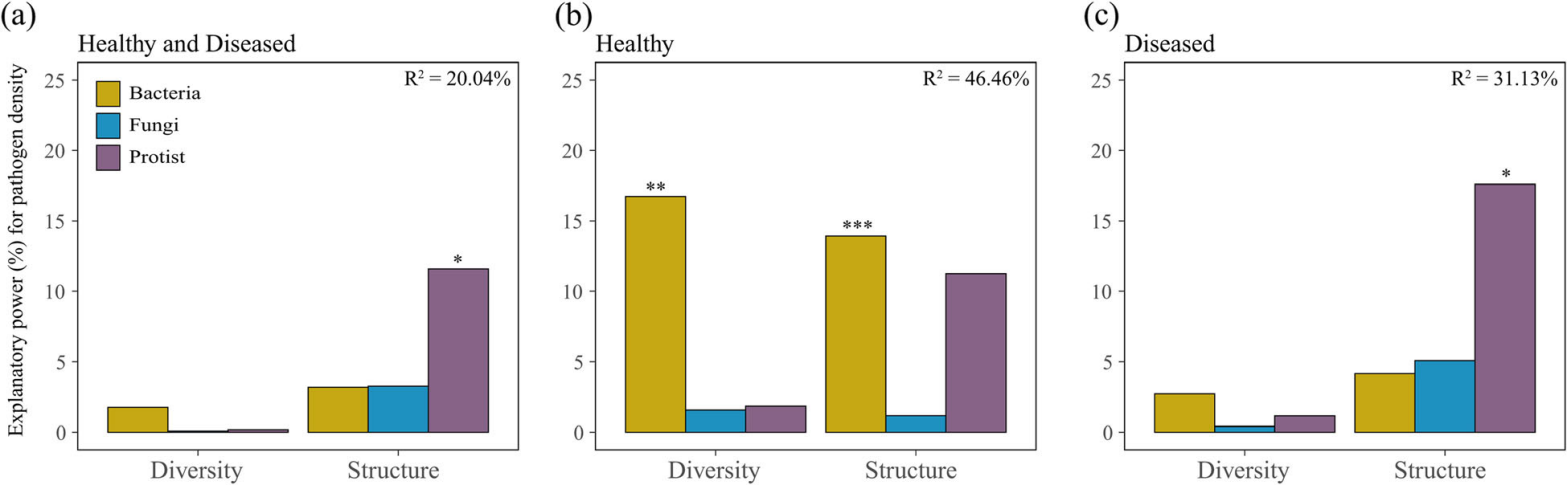
The relative importance of the main microbial parameters in predicting pathogenic *Ralstonia solanacearum* density across plant growth with the combined datasets including healthy and diseased plants (**a**), the healthy plant dataset (**b**), and the diseased plant dataset (**c**). Diversity (Shannon index) and structure (PCoA2) of bacterial, fungal, and protistan communities were selected as the six main microbial predictors (Fig. S2). Asterisk means *P* < 0.05, two asterisks mean *P* < 0.01, and three asterisks mean *P* < 0.001 (statistical significance was calculated by multiple regression using linear models between the microbial predictors and *R. solanacearum*)

**Fig. 2:**
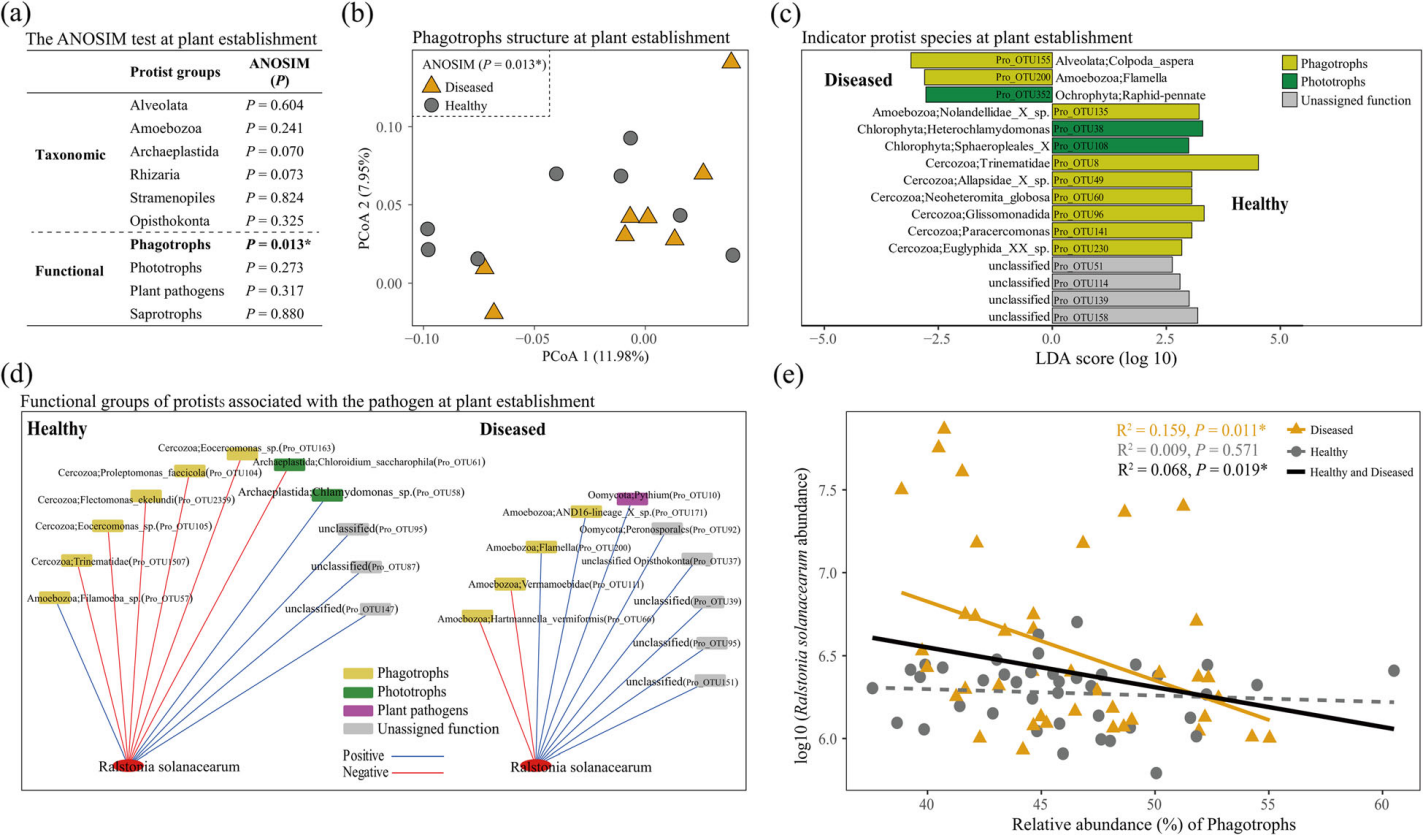
Community structure of protistan taxonomic and functional groups explaining differences between diseased and healthy plants at plant establishment (week 0) (**a**). Community structure of phagotrophic protists (**b**) and indicator protistan OTUs (**c**) in diseased and healthy plants at plant establishment, and networks of the functional groups of protistan OTUs directly associated with the *R. solanacearum* pathogen in healthy and diseased plants at plant establishment (**d**). Correlations between the relative abundance of phagotrophic protists and *R. solanacearum* in diseased and healthy plants across plant growth (**e**). In panel **a**, only abundant taxonomic and functional groups of protists were selected (average relative abundance over 1%). In panel **a** and **b**, asterisk means *P* < 0.05. In panel **c**, protistan OTUs with LDA score > 2.0 are indicators for healthy plants, while protistan OTUs with LDA score < − 2.0 are belonging to diseased plants. In panel **d**, blue lines indicate positive, and red lines indicate negative correlations. In panel **e**, the solid line shows a significant (*P* < 0.05) correlation, and the dashed line shows a non-significant (*P* > 0.05) correlation

Here, we show that the community structure of protists could best predict the density of the *R. solanacearum* pathogen across plant growth in healthy and diseased datasets (Fig. 1a). In healthy plants, the diversity and community structure of bacteria could significantly predict pathogen density (Fig. 1b), which is in line with previous findings that soil bacterial composition can predetermine future plant health [26]. In diseased plants, the community structure of protists was the best predictor for pathogen density (Fig. 1c). At plant establishment, the community structure of bacteria differed (ANOSIM, *P* < 0.001; Table S2) between healthy and diseased plants as shown before [26] but not that of fungi and protists (ANOSIM, *P* > 0.05; Table S2). However, we found that the community structure of phagotrophic protists at plant establishment was indicative for later plant health, as indicated by the differences (ANOSIM, *P* = 0.013) observed between plants developing disease and those remaining healthy (Fig. 2a, b). The community composition of other protistan functional and taxonomic groups did not differ between later healthy or diseased plants at plant establishment (Fig. 2a). Indicator analysis revealed 13 protistan OTUs in healthy plants at plant establishment (with only 3 in diseased plants) that indicate later plant health (Fig. 2c and Table S3). Seven protistan OTUs indicative for healthy plants were identified as phagotrophs, including one amoebozoan and six cercozoan taxa, that likely prey entirely or as part of their diet on bacteria [30]. Of these, the protistan Pro_OTU8 (Cercozoa, Trinematidae) was the most abundant at plant establishment (Table S3) and across plant growth accounting for around 11% of all protistan reads (Table S5). This taxon likely represents an omnivorous protist that mostly feeds on bacteria [30]. Co-occurrence network analysis revealed more negative links between *R. solanacearum* and protistan OTUs in healthy than in diseased plants at plant establishment (Fig. 2d and Table S4). Particularly phagotrophs (a taxon within *Trinematidae*, *Flectomonas ekelundi*, *Proleptomonas faecicola*, and two *Eocercomonas* spp*.*, all mostly bacterivorous Cercozoa) but also a phototrophic *Chloroidium saccharophila* were negatively linked with the pathogen at plant establishment (Fig. 2d). Although those protistan OTUs were also present in diseased plants, they did not correlate with the pathogen in the network analysis (Table S4). Thus, we conclude that phagotrophic protists in general as well as specific taxa at plant establishment can predict pathogen density and plant health at plant maturation, as supported by the community structure of phagotrophs, phagotrophic indicator taxa, and negative links between phagotrophic protistan OTUs and the pathogen in co-occurrence networks. This supports the perspective that functional units rather than taxonomic units underlie microbial functioning and as such should be considered as better indicators [31–33], even across different trophic levels in the microbiome. In addition, we found that the relative abundance of total phagotrophs correlated negatively (regression analysis, *P* < 0.05) with the abundance of *R. solanacearum* in diseased plants or in healthy and diseased combined datasets across plant growth (Fig. 2e). Interestingly, the relative abundance of total phagotrophs significantly decreased (regression analysis, *P* < 0.05) with plant growth time in diseased plants (Fig. S4). Phagotrophic protists may control pathogen development throughout plant growth, as a decreased relative abundance of phagotrophs in diseased plants coincided with pathogen outbreak. Although the pathogen was present in healthy plants, a stable relative abundance of phagotrophic protists throughout plant development might have helped to keep the pathogen in check. Together, these findings suggest that direct trophic interactions between phagotrophic protists and the pathogen at plant establishment and through plant growth steer later plant performance. In contrast, *R. solanacearum* in diseased plants at plant establishment was positively linked with two oomycete species (OTU), including one likely plant-pathogenic *Pythium* species (Fig. 2d). This suggests that a pathobiome forms in diseased plants [34, 35], here consisting of a simultaneous infection with different pathogens. While, a dominance of predator-prey interactions might mitigate negative pathogen effects and thereby stimulating plant health.

**Fig. 3:**
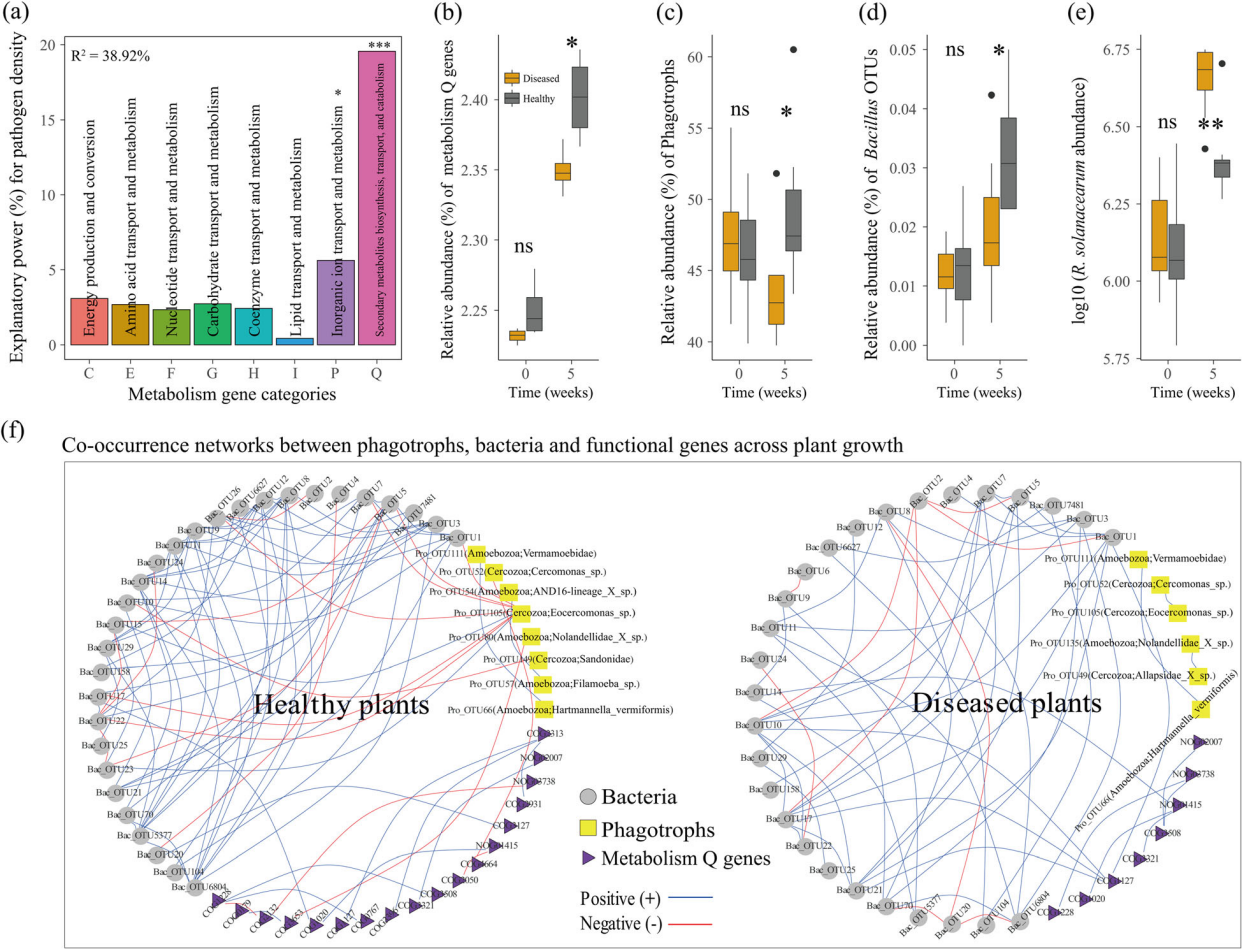
Relative importance of the eight metabolism gene categories in predicting *R. solanacearum* density across plant growth in the combined datasets including healthy and diseased plants (**a**). Changes in relative abundance of metabolism Q genes (secondary metabolite biosynthesis, transport, and catabolism genes) in diseased and healthy plants at week 0 and week 5 (**b**). Relative abundance of phagotrophic protists in diseased and healthy plants at week 0 and week 5 (**c**). Relative abundance of *Bacillus* OTUs in diseased and healthy plants at week 0 and week 5 (**d**). Abundance of *R. solanacearum* in diseased and healthy plants at week 0 and week 5 (**e**). Co-occurrence networks between abundant phagotrophic protistan OTUs, bacterial OTUs, and metabolism Q genes for healthy and diseased plants across plant growth (**f**). In panel **a**, asterisk means *P* < 0.05 and three asterisks mean *P* < 0.001 (statistical significance was calculated by multiple regression using linear models between metabolism genes and *R. solanacearum* pathogen). In panel **b**, **c**, **d,** and **e**, “ns” means non-significant, asterisk means *P* < 0.05 and two asterisks mean *P* < 0.01 under student’s *t* test (*n* = 4 for metabolism Q genes, *n* = 8 for phagotrophic protists, *Bacillus* and *R. solanacearum*). In panel **d**, relative abundance of *Bacillus* OTUs combines the two *Bacillus* OTUs from the bacterial OTU table. In panel **f**, blue lines indicate positive, and red lines indicate negative correlations; detailed annotation of bacterial OTUs and metabolism Q genes are provided in Table S5

We also found that protists might determine pathogen development and plant health through functional changes in the bacterial microbiome. Healthy plants showed significantly (student’s *t* test, *P* < 0.05) higher relative abundances of metabolism genes related with carbohydrate and coenzyme functions at plant establishment (Fig. S5). Strikingly, most metabolism genes had significantly (student’s *t* test, *P* < 0.05) higher relative abundances in healthy than in diseased plants at week 5 (Fig. S5). Among the eight metabolism gene categories, secondary metabolite biosynthesis [Q] genes were most strongly linked (lineal model, *P* < 0.001) with *R. solanacearum* density (Fig. 3a). Furthermore, the relative abundance of metabolism [Q] genes increased over time in healthy plants, showing significantly (student’s *t* test, *P* < 0.05) higher relative abundance in healthy than in diseased plants at week 5 (Fig. 3b). Metabolism [Q] genes did not differ between healthy and diseased plants at weeks 0, 3, and 4 (Fig. S5). Heathy plants with a higher (student’s *t* test, *P* < 0.05) relative abundance of phagotrophic protists (Fig. 3c) had a higher (student’s *t* test, *P* < 0.05) relative abundance of metabolism [Q] genes (Fig. 3b), a higher (student’s *t* test, *P* < 0.05) relative abundance of *Bacillus* OTUs (Fig. 3d), and a lower (student’s *t* test, *P* < 0.01) level of pathogen density than diseased plants at week 5 (Fig. 3e and Fig. S1). In addition, co-occurrence networks encompassing phagotrophic protistan OTUs, bacterial OTUs, and metabolism [Q] genes across plant growth showed that phagotrophs had more correlations with bacteria (9 links with 7 negative) and functional genes (2 links) in healthy plants than in diseased plants (0 links) (Fig. 3f). Especially, Pro_OTU105 (Cercozoa; *Eocercomonas* sp.), which also negatively correlated with the pathogen at plant establishment, showed negative (Spearman’s correlation coefficient (*ρ*) < − 0.8 with *P* < 0.01) correlations with six bacterial OTUs across plant growth. Among those was one bacterial OTU (Bac_OTU17: Bacteroidetes; *Terrimonas*) that positively linked with non-ribosomal peptide synthetase gene (COG1020), one of the key genes involved in the suppression of *R. solanacearum* pathogen [26, 36] (Fig. 3f and Table S5). Future targeted experiments using isolated phagotrophic protists and bacterial strains are needed to evaluate such a role. Healthy plants showed higher numbers of phagotrophic protistan OTUs, bacterial OTUs, and metabolism [Q] genes, resulting in a more complex network (55 nodes with 90 links) than diseased plants (41 nodes with 59 links) (Fig. 3f and Table S6). Specific linkages within co-occurrence networks only provide information about potential interactions, but further mechanistic proof for the interaction needs specific co-culture experiments. In addition to individual links, network structure and composition can provide insights about system’s stability and increased potential for providing ecosystem services [37–40], suggesting that healthy plants benefit from the presence of a more complex network, among them higher numbers of phagotrophs (higher-trophic level organisms in general).

Our findings bridge evidence from laboratory or greenhouse studies focusing on single protist model species [18, 20, 41, 42] to the community level in agricultural systems under field conditions, showing that protists affect bacterial communities and their functioning through predation, leading to changes in plant performance. Compared with diseased plants, healthy plants hosted higher relative abundances of phagotrophic protists, potentially plant-beneficial bacteria, and secondary metabolite genes likely implicated in pathogen suppression 5 weeks after plant establishment—the time point when pathogen symptoms first developed in diseased plants (Fig. S1). Moreover, phagotrophic protists negatively correlated with bacteria that positively linked with a pathogen-suppressing gene coding for non-ribosomal peptides across plant growth. This finding might also contribute to pathogen suppression. However, the interaction between plants and the rhizosphere microbiome is a complex and dynamic process [43]. Future experiments are needed to further examine how plants affect bacterial, fungal, and protistan communities and their interactions and how those changes in the soil microbiome in turn affects plant performance. Together, we propose that predation-induced shifts in microbiome composition and functioning are likely involved in controlling pathogen development and therefore plant health.

## Conclusions

Using a holistic microbiome investigation of bacteria, fungi, and protists in the rhizosphere across plant growth, we show that in addition to bacteria, protists serve as key indicators that predict plant health. Particularly, the community composition of phagotrophic protists during plant establishment can predict later plant performance in the presence of pathogens. These protists might indeed protect plants by directly feeding on the pathogen and through predation-induced shifts in the taxonomic and functional composition of bacteria. These results hold promise in creating tailor-made systems to predict plant performance based on protistan communities before a crop plant is grown. Furthermore, our findings suggest a potential for targeted microbiome engineering to promote plant performance through the application of key microbiome predators: protists. This would bring us closer to the holy grail in reaching a more sustainable, pesticide-reduced agriculture.

## Methods

### Experiment description and soil samples collection

We used a semi-open mesocosm system (rhizobox) as described previously [26], which allowed repeated collection of rhizosphere soil from each individual plant without damaging the root system under field condition. Briefly, each individual tomato plant was grown in a rhizobox filled with the original local soil. Triplicate soil samples were collected from the inner and outer sides of the middle sampling layer, which were thoroughly homogenized and pooled. These soil samples were regarded as the initial bulk soil samples (week 0). In order to track the imprint of the tomato rhizosphere, three nylon bags from each rhizobox were collected 3, 4, 5, and 6 weeks after transplantation. We focused our analyses on the two categories that clearly differed between plants, i.e., plants with wilt symptoms and detectable pathogen (*R. solanacearum*) levels and no wilt symptoms with no detectable pathogen levels. Ten other plants that did not show wilt symptoms, but did have detectable pathogen levels at a later stage (latently infected plants), were not included in further analyses [26]. Soils from the three nylon bags (4 g soil per bag) for each tomato plant at each time point were separately homogenized with sterilized forceps and stored at – 80 °C for further use. Soil DNA was extracted from 0.5 g soil using the MoBioPowerSoil™ DNA Isolation Kit (Mo Bio Laboratories Inc., Carlsbad, CA, USA) according to the manufacturer’s instructions. We used the DNA samples to determine rhizosphere bacterial and eukaryotic communities as well as functional genes in both healthy and diseased tomato plants across plant growth.

### Illumina MiSeq sequencing of the 16S rRNA gene and the 18S rRNA gene

The V4 region of the 16S rRNA gene was PCR-amplified to investigate bacterial communities using the primer set 563F and 802R [44] as described previously [26]. In addition, we selected 80 DNA samples (2 symptoms × 5 time points × 8 replicates) for eukaryotic community profiling. For that, the V4 region of the 18S rRNA gene was broadly targeted to investigate eukaryotes using the primer set V4_1f (CCAGCASCYGCGGTAATWCC) and TAReukREV3 (ACTTTCGTTCTTGATYRA) [45]. PCR was performed in a 20 μl volume consisting of 4 μl of 5× reaction buffer, 2 μl dNTPs (2.5 mM), 0.8 μl of each primer (10 uM), FastPfu Polymerase 0.4 μl, 10 ng of DNA template, and the rest being ddH_2_O. Amplification was performed with the following temperature regime: 5 min of initial denaturation at 95 °C, followed by 30 cycles of denaturation (95 °C for 30 s), annealing (55 °C for 30 s), extension (72 °C for 45 s), and a final extension at 72 °C for 10 min. PCR products were pooled in equimolar concentrations of 10 ng μl^− 1^. Paired-end sequencing was performed on an Illumina MiSeq sequencer at Shanghai Biozeron Biological Technology Co. Ltd (Shanghai, China).

### Bioinformatic analyses of bacteria, fungi, and protist communities

16S rRNA gene sequence data was processed with the UPARSE pipeline as described previously [26]. After removing the reads assigned as chloroplast, mitochondria, and unknown taxa, we obtained 9108 prokaryotic OTUs (9051 bacteria OTUs and 57 archaea OTUs). We further removed archaeal OTUs (accounting for less than 0.05% of total prokaryotic reads) to generate a bacterial OTU table. We selected 8 replicates from the 12 replicates for bacterial community profiles which matched the 80 eukaryotic datasets (2 symptoms × 5 time points × 8 replicates). Each sample from the bacterial OTU table was rarefied to 26,014 reads resulting in 8656 bacterial OTUs. We extracted bacterial OTUs from *Bacillus* and *Pseudomonas* (Fig. 3d and Fig. S6), both well-known potentially biocontrol agents against various soil-borne pathogens including *R. solanacearum* [46–48].

Eukaryotic sequences were processed according to previously established protocols [49, 50] with some modifications. In short, sequences with expected errors > 1.0 or a length shorter than 350 bp were removed. After discarding singletons, the remaining reads were assigned to operational taxonomic units (OTUs) with a 97% similarity threshold, followed by a removal of chimeras using UCHIME [51]. Finally, eukaryotic OTUs were matched against the PR^2^ database [52]. In order to obtain the protistan OTU table, we removed sequences belonging to Rhodophyta, Streptophyta, Metazoa, and Fungi, resulting in 1,475,483 reads for the 80 samples (average 18,444 reads per sample). In order to obtain an equivalent sequencing depth for later analyses, all samples were rarefied to 4537 sequences in 1926 protistan OTUs. We further assigned the protistan OTUs into different functional groups according to their nutrient-uptake mode based on literature [49, 50], including parasites, phagotrophs, phototrophs, plant pathogens, and saprotrophs (Table S1). From the eukaryotic OTU table, we extracted OTUs assigned as fungi resulting in 525,927 reads for the 80 samples (average 6574 reads per sample). Each sample from the fungal OTU table was rarefied to 1085 reads in 234 fungal OTUs.

### Functional genes from meta-genomic sequencing

We had 12 replicates (each time point) for both diseased and healthy plants. We selected 4 of those replicates (40 samples in total: 2 symptoms × 5 time points × 4 replicates) for metagenome analyses. Meta-genomic analysis and functional annotation were performed previously [26]. In short, all reads were trimmed by the Sickle software that removing reads quality below 20 and shorter than 50 bp. Filtered reads were assembled with SOAPdenovo. Assembled contigs were then predicted using MetaGene [53] and clustered with a 0.95 similarity threshold using CD-HIT to generate non-redundant gene catalog. The quality filtered reads from each sample were subsequently mapped to the represent genes using SOAPaligner. Functional gene annotation was carried out against eggNOG database [54]. In order to focus on potentially functional activities of the microbiome in the rhizosphere across plants growth, we extracted microbial metabolism genes (representing 44.85% of all functional genes, Fig. S5), including the following eight general categories: [C] energy production and conversion, [E] amino acid transport and metabolism, [F] nucleotide transport and metabolism, [G] carbohydrate transport and metabolism, [H] coenzyme transport and metabolism, [I] lipid transport and metabolism, [P] inorganic ion transport and metabolism, and [Q] secondary metabolite biosynthesis, transport, and catabolism.

### Co-occurrence network and statistical analyses

First, we used co-occurrence networks to uncover the potential interactions between the functional groups of protists and the pathogen *R. solanacearum* for diseased and healthy plants at each time point. We selected abundant functional groups of protistan OTUs (with average relative abundance > 0.1% across all the samples) and the abundance of *R. solanacearum* for network constructions. Second, we used the co-occurrence networks to uncover potential interactions between phagotrophic protists, bacteria, and functional genes for diseased and healthy plants across plant growth (combined all time point samples in diseased or heathy plants). As we selected 8 replicates from the 12 replicates for eukaryotic community profiles with 3 metagenomic replicates matching both bacterial and eukaryotic datasets, we used the 30 samples in total (2 symptoms × 5 time points × 3 replicates) for the analyses. We further selected abundant phagotrophic protistan OTUs (top 30), bacterial OTUs (top 30), and metabolism Q genes (top 30 genes in metabolism Q category) for network constructions (detailed information provided in Table S5). A pairwise Spearman correlation matrix was calculated with the “corr.test” function in the package “psych” in R (version 3.4.4). The *P* values were adjusted with the false discovery rate method [55]. Spearman’s correlation coefficient (*ρ*) higher than 0.7 (or lower than − 0.7) with *P* values < 0.05 was selected for the networks of “functional groups of protistan OTUs with the *R. solanacearum*.” In order to select robust correlations between phagotrophic protistan OTUs, bacterial OTUs, and metabolism Q genes, Spearman’s correlation coefficients (*ρ*) higher than 0.8 (or lower than − 0.8) with *P* values < 0.01 were selected for the network of “phagotrophic protists, bacteria, and functional genes”. Network properties were characterized via the “igraph” package in R (version 3.4.4). Finally, networks of “functional groups of protists directly associated with the *R. solanacearum*” at plant establishment for healthy and diseased plants were visualized in Cytoscape (v3.5.1), and co-occurrence networks of “phagotrophic protistan OTUs, bacterial OTUs, and metabolism Q genes” for healthy and diseased plants were visualized via the “igraph” package in R (version 3.4.4).

The α-diversity of bacterial, fungal, and protistan communities across plant growth was estimated using the non-parametric Shannon index [56]. A principal coordinate analysis (PCoA) based on Bray–Curtis distance metrics was performed in R (version 3.4.4) to explore the differences in bacterial, fungal, and protist community structures (Hellinger transformed) across plant growth. ANOSIM was applied to investigate significant differences of microbial community structures between diseased and healthy plants at each time point. Abundant protistan OTUs (average relative abundance > 0.1%) were used to examine indicator species, which were assessed in LEfSe [57] through the “lefse command” in Mothur [58]. In addition, we used the “relaimpo” package [59] in R (version 3.4.4) to calculate the relative importance of main microbial parameters in predicting *R. solanacearum* density across plant growth in the combined dataset including healthy and diseased plants, healthy plant dataset, and diseased plant dataset. We selected the diversity (Shannon index) and structure (PCoA2) of bacteria, fungi, and protists as the six main microbial predictors (Fig. S2) and used multiple regression by lineal models in R (version 3.4.4) to calculate the significance of the correlation between microbial predictors and *R. solanacearum* (all data was standardized by “scale” function in R). We also used the “relaimpo” package to calculate the relative importance of the eight metabolism genes for *R. solanacearum* density across plant growth in the combined healthy and diseased plant samples. Other linear regression relationships were examined by the “lm” function in R (version 3.4.4). Student’s *t* test was used to compare the microbial taxon and functional gene differences between diseased and healthy plants at each time point. Normal distribution was tested by the Shapiro-Wilk test; non-normal data were log or log (x + 1) transformed [60].

## Supplementary information

**Additional file 1: Table S1.** Annotation table for assigning protistan functional groups.

**Additional file 2: Table S2.** Differences (based on ANOSIM) in the community structure of bacteria, fungi and protists (including the abundant taxonomic and functional groups of protists) between diseased and healthy plants at each time point across plant growth. **Table S3.** Detailed information of indicator protistan taxa (OTUs) in diseased and healthy plants at plant establishment. **Table S4.** Detailed information of protistan taxa (OTUs) in the networks of “functional groups of protists directly associated with the *R. solanacearum* pathogen” in diseased and healthy plants at plant establishment. **Table S5.** Detailed information of the 30 most abundant phagotrophic protistan OTUs, bacterial OTUs and metabolism Q genes in the co-occurrence networks shown in Fig. 3f. **Table S6.** Topological properties of networks between the abundant phagotrophic protistan OTUs, bacterial OTUs and metabolism Q genes for healthy and diseased plants across plant growth. **Figure S1.** Abundance of *R. solanacearum* pathogen in diseased and healthy plants across plant growth. **Figure S2.** Shannon diversity of bacteria (a), fungi (b) and protists (c) in diseased and healthy plants across plant growth, and overall community structures of bacteria (d), fungi (e) and protists (f) in diseased and healthy plants across plant growth. **Figure S3.** Relative abundances of the most abundant (average relative abundance over 1% across all samples) taxonomic (a, b, c, d, e and f) and functional (g, h, i, j and k) groups of protists in diseased and healthy plants across plant growth. **Figure S4.** Correlation between the relative abundance of phagotrophic protists and plant growth time in diseased and healthy plants. **Figure S5.** Relative abundances of the eight metabolism genes and total metabolism genes in diseased and healthy plants across plant growth. **Figure S6.** Relative abundance of *Pseudomonas* OTUs in diseased and healthy plants at week 0 and week 5.

## Data Availability

All raw 16S rRNA gene sequence data is available at the DDBJ Sequence Read Archive (DRA) under the accession number SRP090147. All raw 18S rRNA gene sequences are available at the NCBI Sequence Read Archive (SRA) under the accession number PRJNA525676. The raw data of metagenomics-derived gene catalogs are publicly available under the accession number PRJNA492172.

## References

[1] Dean R (2012). Kan J a. LV, Pretorius ZA, Hammond-Kosack KE, Pietro AD, Spanu PD, et al. The top 10 fungal pathogens in molecular plant pathology. Mol Plant Pathol.

[2] Mansfield J, Genin S, Magori S, Citovsky V, Sriariyanum M, Ronald P (2012). Top 10 plant pathogenic bacteria in molecular plant pathology. Mol Plant Pathol.

[3] Berendsen RL, Pieterse CMJ, Bakker PAHM (2012). The rhizosphere microbiome and plant health. Trends Plant Sci.

[4] Mendes R, Garbeva P, Raaijmakers JM (2013). The rhizosphere microbiome: significance of plant beneficial, plant pathogenic, and human pathogenic microorganisms. FEMS Microbiol Rev.

[5] Philippot L, Raaijmakers JM, Lemanceau P, van der Putten WH (2013). Going back to the roots: the microbial ecology of the rhizosphere. Nat Rev Microbiol.

[6] Sapp M, Ploch S, Fiore-Donno AM, Bonkowski M, Rose LE (2018). Protists are an integral part of the Arabidopsis thaliana microbiome. Environ Microbiol.

[7] Vorholt JA, Vogel C, Carlström CI, Müller DB (2017). Establishing causality: opportunities of synthetic communities for plant microbiome research. Cell Host Microbe.

[8] Gao Z, Karlsson I, Geisen S, Kowalchuk G, Jousset A (2019). Protists: puppet masters of the rhizosphere microbiome. Trends Plant Sci.

[9] Cha J-Y, Han S, Hong H-J, Cho H, Kim D, Kwon Y (2016). Microbial and biochemical basis of a Fusarium wilt-suppressive soil. ISME J.

[10] Mendes R, Kruijt M, de Bruijn I, Dekkers E, van der Voort M, Schneider JHM (2011). Deciphering the rhizosphere microbiome for disease-suppressive bacteria. Science.

[11] Sanguin H, Sarniguet A, Gazengel K, Moënne-Loccoz Y, Grundmann GL (2009). Rhizosphere bacterial communities associated with disease suppressiveness stages of take-all decline in wheat monoculture. New Phytol.

[12] Manici LM, Caputo F (2009). Fungal community diversity and soil health in intensive potato cropping systems of the east Po valley, Northern Italy. Ann Appl Biol.

[13] Penton CR, Gupta V, Tiedje JM, Neate SM, Ophel-Keller K, Gillings M (2014). Fungal community structure in disease suppressive soils assessed by 28S LSU gene sequencing. PLoS One.

[14] Hassani MA, Durán P, Hacquard S (2018). Microbial interactions within the plant holobiont. Microbiome.

[15] Adl MS, Gupta VS (2006). Protists in soil ecology and forest nutrient cycling. Can J For Res.

[16] Geisen S, Mitchell EAD, Adl S, Bonkowski M, Dunthorn M, Ekelund F (2018). Soil protists: a fertile frontier in soil biology research. FEMS Microbiol Rev.

[17] Henkes GJ, Kandeler E, Marhan S, Scheu S, Bonkowski M (2018). Interactions of mycorrhiza and protists in the rhizosphere systemically alter microbial community composition, plant shoot-to-root ratio and within-root system nitrogen allocation. Front Environ Sci.

[18] Rosenberg K, Bertaux J, Krome K, Hartmann A, Scheu S, Bonkowski M (2009). Soil amoebae rapidly change bacterial community composition in the rhizosphere of Arabidopsis thaliana. ISME J.

[19] Geisen S, Koller R, Hünninghaus M, Dumack K, Urich T, Bonkowski M (2016). The soil food web revisited: diverse and widespread mycophagous soil protists. Soil Biol Biochem.

[20] Bonkowski M (2004). Protozoa and plant growth: the microbial loop in soil revisited. New Phytol.

[21] Schulz-Bohm K, Geisen S, Wubs ERJ, Song C, de Boer W, Garbeva P (2017). The prey’s scent – volatile organic compound mediated interactions between soil bacteria and their protist predators. ISME J.

[22] Glücksman E, Bell T, Griffiths RI, Bass D (2010). Closely related protist strains have different grazing impacts on natural bacterial communities. Environ Microbiol.

[23] Jousset A, Lara E, Wall LG, Valverde C (2006). Secondary metabolites help biocontrol strain pseudomonas fluorescens CHA0 to escape protozoan grazing. Appl Environ Microbiol.

[24] Mazzola M, de Bruijn I, Cohen MF, Raaijmakers JM (2009). Protozoan-induced regulation of cyclic lipopeptide biosynthesis is an effective predation defense mechanism for Pseudomonas fluorescens. Appl Environ Microbiol.

[25] Hünninghaus M, Dibbern D, Kramer S, Koller R, Pausch J, Schloter-Hai B (2019). Disentangling carbon flow across microbial kingdoms in the rhizosphere of maize. Soil Biol Biochem.

[26] Wei Z, Gu Y, Friman V-P, Kowalchuk GA, Xu Y, Shen Q (2019). Initial soil microbiome composition and functioning predetermine future plant health. Sci Adv.

[27] Zhao Z-B, He J-Z, Geisen S, Han L-L, Wang J-T, Shen J-P (2019). Protist communities are more sensitive to nitrogen fertilization than other microorganisms in diverse agricultural soils. Microbiome.

[28] Jiang G, Wei Z, Xu J, Chen H, Zhang Y, She X (2017). Bacterial wilt in China: history, current status, and future perspectives. Front Plant Sci.

[29] Salanoubat M, Genin S, Artiguenave F, Gouzy J, Mangenot S, Arlat M (2002). Genome sequence of the plant pathogen Ralstonia solanacearum. Nature.

[30] Dumack K, Fiore-Donno AM, Bass D, Bonkowski M. Making sense of environmental sequencing data: ecologically important functional traits of the protistan groups Cercozoa and Endomyxa (Rhizaria). Mol Ecol Resour. 2019; Available from: https://onlinelibrary.wiley.com/doi/abs/10.1111/1755-0998.13112. 10.1111/1755-0998.13112 31677344

[31] Burke C, Steinberg P, Rusch D, Kjelleberg S, Thomas T (2011). Bacterial community assembly based on functional genes rather than species. Proc Natl Acad Sci.

[32] Louca S, Parfrey LW, Doebeli M (2016). Decoupling function and taxonomy in the global ocean microbiome. Science.

[33] Ma X, Zhang Q, Zheng M, Gao Y, Yuan T, Hale L (2019). Microbial functional traits are sensitive indicators of mild disturbance by lamb grazing. ISME J.

[34] Bass D, Stentiford GD, Wang H-C, Koskella B (2019). Tyler CR.

[35] Vayssier-Taussat M, Albina E, Citti C, Cosson JF, Jacques M-A, Lebrun M-H (2014). Shifting the paradigm from pathogens to pathobiome: new concepts in the light of meta-omics. Front Cell Infect Microbiol.

[36] Michelsen CF, Watrous J, Glaring MA, Kersten R, Koyama N, Dorrestein PC (2015). Nonribosomal peptides, key biocontrol components for Pseudomonas fluorescens In5, Isolated from a Greenlandic Suppressive Soil. mBio.

[37] Morriën E, Hannula SE, Snoek LB, Helmsing NR, Zweers H, de Hollander M (2017). Soil networks become more connected and take up more carbon as nature restoration progresses. Nat Commun.

[38] de Araujo ASF, Mendes LW, Lemos LN, Antunes JEL, Beserra JEA (2018). Lyra M do CCP de, et al. Protist species richness and soil microbiome complexity increase towards climax vegetation in the Brazilian Cerrado. Commun Biol.

[39] Montoya JM, Rodríguez MA, Hawkins BA (2003). Food web complexity and higher-level ecosystem services. Ecol Lett.

[40] Soliveres S, van der Plas F, Manning P, Prati D, Gossner MM, Renner SC (2016). Biodiversity at multiple trophic levels is needed for ecosystem multifunctionality. Nature.

[41] Flues S, Bass D, Bonkowski M (2017). Grazing of leaf-associated Cercomonads (Protists: Rhizaria: Cercozoa) structures bacterial community composition and function. Environ Microbiol.

[42] Kreuzer K, Adamczyk J, Iijima M, Wagner M, Scheu S, Bonkowski M (2006). Grazing of a common species of soil protozoa (Acanthamoeba castellanii) affects rhizosphere bacterial community composition and root architecture of rice (Oryza sativa L.). Soil Biol Biochem.

[43] Shi S, Nuccio EE, Shi ZJ, He Z, Zhou J, Firestone MK (2016). The interconnected rhizosphere: high network complexity dominates rhizosphere assemblages. Ecol Lett.

[44] Cardenas E, Wu W-M, Leigh MB, Carley J, Carroll S, Gentry T (2010). Significant association between sulfate-reducing bacteria and uranium-reducing microbial communities as revealed by a combined massively parallel sequencing-indicator species approach. Appl Environ Microbiol.

[45] Bass D, Silberman JD, Brown MW, Pearce RA, Tice AK, Jousset A (2016). Coprophilic amoebae and flagellates, including Guttulinopsis, Rosculus and Helkesimastix, characterise a divergent and diverse rhizarian radiation and contribute to a large diversity of faecal-associated protists. Environ Microbiol.

[46] Raaijmakers JM, De Bruijn I, Nybroe O, Ongena M (2010). Natural functions of lipopeptides from Bacillus and Pseudomonas: more than surfactants and antibiotics. FEMS Microbiol Rev.

[47] Raza W, Ling N, Liu D, Wei Z, Huang Q, Shen Q (2016). Volatile organic compounds produced by Pseudomonas fluorescens WR-1 restrict the growth and virulence traits of Ralstonia solanacearum. Microbiol Res.

[48] Chen MC, Wang JP, Zhu YJ, Liu B, Yang WJ, Ruan CQ (2019). Antibacterial activity against Ralstonia solanacearum of the lipopeptides secreted from the Bacillus amyloliquefaciens strain FJAT-2349. J Appl Microbiol.

[49] Xiong W, Jousset A, Guo S, Karlsson I, Zhao Q, Wu H (2018). Soil protist communities form a dynamic hub in the soil microbiome. ISME J.

[50] Xiong W, Li R, Guo S, Karlsson I, Jiao Z, Xun W (2019). Microbial amendments alter protist communities within the soil microbiome. Soil Biol Biochem.

[51] Edgar RC, Haas BJ, Clemente JC, Quince C, Knight R (2011). UCHIME improves sensitivity and speed of chimera detection. Bioinformatics.

[52] Guillou L, Bachar D, Audic S, Bass D, Berney C, Bittner L (2013). The Protist Ribosomal Reference database (PR2): a catalog of unicellular eukaryote small sub-unit rRNA sequences with curated taxonomy. Nucleic Acids Res.

[53] Noguchi H, Park J, Takagi T (2006). MetaGene: prokaryotic gene finding from environmental genome shotgun sequences. Nucleic Acids Res.

[54] Huerta-Cepas J, Szklarczyk D, Heller D, Hernández-Plaza A, Forslund SK, Cook H (2019). eggNOG 5.0: a hierarchical, functionally and phylogenetically annotated orthology resource based on 5090 organisms and 2502 viruses. Nucleic Acids Res.

[55] Benjamini Y, Hochberg Y (1995). Controlling the false discovery rate: a practical and powerful approach to multiple testing. J R Stat Soc Ser B Methodol.

[56] Chao A, Shen T-J (2003). Nonparametric estimation of Shannon’s index of diversity when there are unseen species in sample. Environ Ecol Stat.

[57] Segata N, Izard J, Waldron L, Gevers D, Miropolsky L, Garrett WS (2011). Metagenomic biomarker discovery and explanation. Genome Biol.

[58] Schloss PD, Westcott SL, Ryabin T, Hall JR, Hartmann M, Hollister EB (2009). Introducing mothur: open-source, platform-independent, community-supported software for describing and comparing microbial communities. Appl Environ Microbiol.

[59] Groemping U (2006). Relative importance for linear regression in R: the package relaimpo. J Stat Softw.

[60] McDonald JH (2009). Handbook of biological statistics.

