# Supplementary material for: Rhizosphere protists are key determinants of plant health
Source: Microbiome. 2020 Mar 3;8:27. doi: 10.1186/s40168-020-00799-9 (PMC7055055; doi:10.1186/s40168-020-00799-9)
Supplement: Supplementary file 3 — Additional file 2: Table S2. Differences (based on ANOSIM) in the community structure of bacteria, fungi and protists (including the abundant taxonomic and functional groups of protists) between diseased and healthy plants at each time point across plant growth. Table S3. Detailed information of indicator protistan taxa (OTUs) in diseased and healthy plants at plant establishment. Table S4. Detailed information of protistan taxa (OTUs) in the networks of “functional groups of protists directly associated with the R. solanacearum pathogen” in diseased and healthy plants at plant establishment. Table S5. Detailed information of the 30 most abundant phagotrophic protistan OTUs, bacterial OTUs and metabolism Q genes in the co-occurrence networks shown in Fig. 3f. Table S6. Topological properties of networks between the abundant phagotrophic protistan OTUs, bacterial OTUs and metabolism Q genes for healthy and diseased plants across plant growth. Figure S1. Abundance of R. solanacearum pathogen in diseased and healthy plants across plant growth. Figure S2. Shannon diversity of bacteria (a), fungi (b) and protists (c) in diseased and healthy plants across plant growth, and overall community structures of bacteria (d), fungi (e) and protists (f) in diseased and healthy plants across plant growth. Figure S3. Relative abundances of the most abundant (average relative abundance over 1% across all samples) taxonomic (a, b, c, d, e and f) and functional (g, h, i, j and k) groups of protists in diseased and healthy plants across plant growth. Figure S4. Correlation between the relative abundance of phagotrophic protists and plant growth time in diseased and healthy plants. Figure S5. Relative abundances of the eight metabolism genes and total metabolism genes in diseased and healthy plants across plant growth. Figure S6. Relative abundance of Pseudomonas OTUs in diseased and healthy plants at week 0 and week 5. [file 40168_2020_799_MOESM2_ESM.docx]

**Table S2. Differences (based on ANOSIM) in the community structure of bacteria, fungi and protists (including the abundant taxonomic and functional groups of protists) between diseased and healthy plants at each time point across plant growth.**

| **Time**  **Taxon** | **0 week** | **3 weeks** | **4 weeks** | **5 weeks** | **6 weeks** |
| --- | --- | --- | --- | --- | --- |
| **Bacteria** | **R = 0.527,**  ***P* < 0.001***** | R = 0.056,  *P* = 0.222 | R = 0.124,  *P* = 0.106 | R = 0.097,  *P* = 0.110 | **R = 0.215,**  ***P* = 0.037*** |
| **Fungi** | R = -0.023,  *P* = 0.598 | R = 0.155,  *P* = 0.075 | R = -0.047,  *P* = 0.806 | R = 0.095,  *P* = 0.061 | R = 0.080,  *P* = 0.156 |
| **Protists** | R = 0.077,  *P* = 0.112 | R = 0.137,  *P* = 0.079 | R = -0.039,  *P* = 0.692 | R = 0.145,  *P* = 0.052 | R = 0.143,  *P* = 0.064 |
| Alveolata | R = -0.028,  *P* = 0.604 | R = 0.065,  *P* = 0.213 | R = -0.050,  *P* = 0.669 | R = 0.003,  *P* = 0.048 | R = -0.138,  *P* = 0.971 |
| Amoebozoa | R = 0.047,  *P* = 0.241 | R = 0.074,  *P* = 0.177 | R = 0.033,  *P* = 0.265 | **R = 0.124,**  ***P* = 0.023*** | **R = 0.204,**  ***P* = 0.011*** |
| Archaeplastida | R = 0.128,  *P* = 0.070 | R = 0.085,  *P* = 0.145 | R = -0.024,  *P* = 0.627 | R = 0.101,  *P* = 0.124 | **R = 0.198,**  ***P* = 0.016*** |
| Rhizaria | R = 0.099,  *P* = 0.073 | R = 0.008,  *P* = 0.448 | R = -0.033,  *P* = 0.641 | R = -0.029,  *P* = 0.624 | **R = 0.233,**  ***P* = 0.009**** |
| Stramenopiles | R = -0.071,  *P* = 0.824 | R = 0.009,  *P* = 0.414 | R = 0.044,  *P* = 0.237 | R = 0.071,  *P* = 0.186 | R = -0.061,  *P* = 0.765 |
| Opisthokonta | R = 0.026,  *P* = 0.325 | R = 0.166,  *P* = 0.062 | R = -0.067,  *P* = 0.846 | R = 0.041,  *P* = 0.303 | R = -0.077,  *P* = 0.802 |
| Phagotrophs | **R = 0.145,**  ***P* = 0.013*** | R = 0.114,  *P* = 0.106 | R = -0.056,  *P* = 0.775 | R = 0.143,  *P* = 0.066 | R = 0.084,  *P* = 0.143 |
| Phototrophs | R = 0.042,  *P* = 0.273 | **R = 0.156,**  ***P* = 0.043*** | R = -0.040,  *P* = 0.767 | R = 0.050,  *P* = 0.253 | R = -0.004,  *P* = 0.508 |
| Plant pathogens  (Protist function group) | R = 0.019,  *P* = 0.317 | R = -0.054,  *P* = 0.965 | R = -0.004,  *P* = 0.429 | R = 0.021,  *P* = 0.305 | R = -0.015,  *P* = 0.535 |
| Saprotrophs | R = -0.088,  *P* = 0.88 | R = -0.118,  *P* = 0.923 | R = -0.083,  *P* = 0.857 | R = 0.001,  *P* = 0.442 | R = 0.039,  *P* = 0.264 |

Only the abundant taxonomic and functional protistan groups with average relative abundance over 1% were selected. *means *P* < 0.05; **means *P* < 0.01; and *** means *P* < 0.001.

**Table S3. Detailed information of indicator protistan taxa (OTUs) in diseased and healthy plants at plant establishment.**

| OTUid | Symptoms | RA (%) in diseased plants | RA (%) in Healthy plants | Function annotation | Taxonomy annotation |
| --- | --- | --- | --- | --- | --- |
| Pro_OTU155 | Diseased | 0.198 | 0.083 | Phagotrophs | Eukaryota(100);Alveolata(100);Ciliophora(100);Colpodea(100);Colpodida(100);Colpodidae(100);Colpoda(96);Colpoda_aspera(91); |
| Pro_OTU200 | Diseased | 0.171 | 0.091 | Phagotrophs | Eukaryota(100);Amoebozoa(100);Conosa(100);Variosea(100);ATCC50593-Flamella-WIM80-lineage(100);Flamella-lineage(100);Flamella(90);unclassified; |
| Pro_OTU352 | Diseased | 0.207 | 0.066 | Phototrophs | Eukaryota(100);Stramenopiles(100);Ochrophyta(100);Bacillariophyta(100);Bacillariophyta_X(100);Raphid-pennate(97);unclassified;unclassified; |
| Pro_OTU135 | Healthy | 0.256 | 0.408 | Phagotrophs | Eukaryota(100);Amoebozoa(100);Lobosa(100);Tubulinea(100);Nolandida(100);Nolandellidae(100);Nolandellidae_X(100);Nolandellidae_X_sp.(100); |
| Pro_OTU38 | Healthy | 0.331 | 0.504 | Phototrophs | Eukaryota(100);Archaeplastida(100);Chlorophyta(100);Chlorophyceae(100);Chlamydomonadales(100);Chlamydomonadales_X(100);Heterochlamydomonas(100);unclassified; |
| Pro_OTU108 | Healthy | 0.061 | 0.231 | Phototrophs | Eukaryota(100);Archaeplastida(100);Chlorophyta(100);Chlorophyceae(100);Sphaeropleales(99);Sphaeropleales_X(99);unclassified;unclassified; |
| Pro_OTU8 | Healthy | 7.929 | 10.932 | Phagotrophs | Eukaryota(100);Rhizaria(100);Cercozoa(100);Filosa-Imbricatea(100);Euglyphida(100);Trinematidae(100);unclassified;unclassified; |
| Pro_OTU49 | Healthy | 0.333 | 0.463 | Phagotrophs | Eukaryota(100);Rhizaria(100);Cercozoa(100);Filosa-Sarcomonadea(100);Glissomonadida(100);Allapsidae(100);Allapsidae_X(93);Allapsidae_X_sp.(93); |
| Pro_OTU60 | Healthy | 0.240 | 0.355 | Phagotrophs | Eukaryota(100);Rhizaria(100);Cercozoa(100);Filosa-Sarcomonadea(100);Glissomonadida(100);Sandonidae(100);Neoheteromita(97);Neoheteromita_globosa(85); |
| Pro_OTU96 | Healthy | 0.430 | 0.515 | Phagotrophs | Eukaryota(100);Rhizaria(100);Cercozoa(100);Filosa-Sarcomonadea(92);Glissomonadida(92);unclassified;unclassified;unclassified; |
| Pro_OTU141 | Healthy | 0.072 | 0.124 | Phagotrophs | Eukaryota(100);Rhizaria(100);Cercozoa(100);Filosa-Sarcomonadea(100);Cercomonadida(100);Paracercomonadidae(99);Paracercomonas(99);unclassified; |
| Pro_OTU230 | Healthy | 0.061 | 0.129 | Phagotrophs | Eukaryota(100);Rhizaria(100);Cercozoa(100);Filosa-Imbricatea(98);Euglyphida(98);Euglyphida_X(98);Euglyphida_XX(98);Euglyphida_XX_sp.(98); |
| Pro_OTU51 | Healthy | 0.477 | 0.636 | Unassigned | Eukaryota(100);unclassified;unclassified;unclassified;unclassified;unclassified;unclassified;unclassified; |
| Pro_OTU114 | Healthy | 0.143 | 0.198 | Unassigned | Eukaryota(100);unclassified;unclassified;unclassified;unclassified;unclassified;unclassified;unclassified; |
| Pro_OTU139 | Healthy | 0.069 | 0.149 | Unassigned | Eukaryota(100);unclassified;unclassified;unclassified;unclassified;unclassified;unclassified;unclassified; |
| Pro_OTU158 | Healthy | 0.030 | 0.152 | Unassigned | Eukaryota(100);unclassified;unclassified;unclassified;unclassified;unclassified;unclassified;unclassified; |

RA = average relative abundance of the 8 replicates for diseased plants or healthy plants at plant establishment.

**Table S4. Detailed information of protistan taxa (OTUs) in the networks of “functional groups of protists directly associated with the *R. solanacearum* pathogen” in diseased and healthy plants at plant establishment.**

| OTUid | Symptoms | RA (%) in diseased plants | RA (%) in Healthy plants | Function annotation | Taxonomy annotation |
| --- | --- | --- | --- | --- | --- |
| Pro_OTU10 | Diseased | 3.006 | 2.642 | Plant pathogens | Eukaryota(100);Stramenopiles(100);Stramenopiles_X(100);Oomycota(100);Oomycota_X(100);Peronosporales(100);Pythium(100);unclassified; |
| Pro_OTU66 | Diseased | 0.920 | 1.278 | Phagotrophs | Eukaryota(100);Amoebozoa(100);Lobosa(100);Tubulinea(100);Echinamoebida(100);Vermamoebidae(100);Hartmannella(100);Hartmannella_vermiformis(100); |
| Pro_OTU37 | Diseased | 0.774 | 0.678 | Unassigned | Eukaryota(100);Opisthokonta(94);unclassified;unclassified;unclassified;unclassified;unclassified;unclassified; |
| Pro_OTU39 | Diseased | 0.625 | 0.661 | Unassigned | Eukaryota(100);unclassified;unclassified;unclassified;unclassified;unclassified;unclassified;unclassified; |
| Pro_OTU111 | Diseased | 0.358 | 0.419 | Phagotrophs | Eukaryota(100);Amoebozoa(100);Lobosa(100);Tubulinea(100);Echinamoebida(100);Vermamoebidae(100);unclassified;unclassified; |
| Pro_OTU95 | Diseased | 0.284 | 0.226 | Unassigned | Eukaryota(100);unclassified;unclassified;unclassified;unclassified;unclassified;unclassified;unclassified; |
| Pro_OTU92 | Diseased | 0.055 | 0.008 | Unassigned | Eukaryota(100);Stramenopiles(100);Stramenopiles_X(100);Oomycota(100);Oomycota_X(100);Peronosporales(100);unclassified;unclassified; |
| Pro_OTU200 | Diseased | 0.171 | 0.091 | Phagotrophs | Eukaryota(100);Amoebozoa(100);Conosa(100);Variosea(100);ATCC50593-Flamella-WIM80-lineage(100);Flamella-lineage(100);Flamella(90);unclassified; |
| Pro_OTU171 | Diseased | 0.105 | 0.080 | Phagotrophs | Eukaryota(100);Amoebozoa(95);Conosa(94);Variosea(93);Variosea_X(93);AND16-lineage(87);AND16-lineage_X(87);AND16-lineage_X_sp.(87); |
| Pro_OTU151 | Diseased | 0.105 | 0.083 | Unassigned | Eukaryota(100);unclassified;unclassified;unclassified;unclassified;unclassified;unclassified;unclassified; |
| Pro_OTU1507 | Healthy | 0.810 | 0.865 | Phagotrophs | Eukaryota(100);Rhizaria(100);Cercozoa(100);Filosa-Imbricatea(100);Euglyphida(100);Trinematidae(100);unclassified;unclassified; |
| Pro_OTU57 | Healthy | 0.587 | 0.758 | Phagotrophs | Eukaryota(100);Amoebozoa(100);Conosa(100);Variosea(100);Variosea_X(100);Filamoebidae(100);Filamoeba(100);Filamoeba_sp.(100); |
| Pro_OTU105 | Healthy | 0.653 | 0.614 | Phagotrophs | Eukaryota(100);Rhizaria(100);Cercozoa(100);Filosa-Sarcomonadea(100);Cercomonadida(100);Cercomonadidae(100);Eocercomonas(100);Eocercomonas_sp.(100); |
| Pro_OTU58 | Healthy | 0.273 | 0.405 | Phototrophs | Eukaryota(100);Archaeplastida(100);Chlorophyta(100);Chlorophyceae(100);Chlamydomonadales(97);Chlamydomonadales_X(97);Chlamydomonas(95);Chlamydomonas_sp.(94); |
| Pro_OTU61 | Healthy | 0.127 | 0.163 | Phototrophs | Eukaryota(100);Archaeplastida(100);Chlorophyta(100);Trebouxiophyceae(100);Watanabea-Clade(100);Watanabea-Clade_X(100);Chloroidium(100);Chloroidium_saccharophila(98); |
| Pro_OTU95 | Healthy | 0.284 | 0.226 | Unassigned | Eukaryota(100);unclassified;unclassified;unclassified;unclassified;unclassified;unclassified;unclassified; |
| Pro_OTU2359 | Healthy | 0.366 | 0.256 | Phagotrophs | Eukaryota(100);Rhizaria(100);Cercozoa(100);Filosa-Sarcomonadea(100);Glissomonadida(100);Sandonidae(100);Flectomonas(100);Flectomonas_ekelundi(100); |
| Pro_OTU87 | Healthy | 0.229 | 0.212 | Unassigned | Eukaryota(100);unclassified;unclassified;unclassified;unclassified;unclassified;unclassified;unclassified; |
| Pro_OTU104 | Healthy | 0.300 | 0.317 | Phagotrophs | Eukaryota(100);Rhizaria(100);Cercozoa(100);Filosa-Sarcomonadea(100);Glissomonadida(100);Proleptomonadidae(100);Proleptomonas(100);Proleptomonas_faecicola(100); |
| Pro_OTU147 | Healthy | 0.165 | 0.165 | Unassigned | Eukaryota(100);unclassified;unclassified;unclassified;unclassified;unclassified;unclassified;unclassified; |
| Pro_OTU163 | Healthy | 0.276 | 0.306 | Phagotrophs | Eukaryota(100);Rhizaria(100);Cercozoa(100);Filosa-Sarcomonadea(100);Cercomonadida(100);Cercomonadidae(100);Eocercomonas(100);Eocercomonas_sp.(100); |

RA = average relative abundance of the 8 replicates for diseased plants or healthy plants at plant establishment.

**Table S5. Detailed information of the 30 most abundant phagotrophic protistan OTUs, bacterial OTUs and metabolism Q genes in the co-occurrence networks shown in Fig. 3f.**

| **Id** | **Group** | **RA (%)** | **Annotation** |
| --- | --- | --- | --- |
| Pro_OTU8 | Protist | 10.835 | Eukaryota(100);Rhizaria(100);Cercozoa(100);Filosa-Imbricatea(100);Euglyphida(100);Trinematidae(100);unclassified;unclassified; |
| Pro_OTU22 | Protist | 5.559 | Eukaryota(100);Alveolata(100);Ciliophora(100);Spirotrichea(100);Hypotrichia(100);Oxytrichidae(93);Oxytrichidae_X(93);Oxytrichidae_X_sp.(93); |
| Pro_OTU35 | Protist | 1.462 | Eukaryota(100);Alveolata(100);Ciliophora(100);Spirotrichea(100);Hypotrichia(100);Oxytrichidae(100);Gastrostyla(100);Gastrostyla_steinii(100); |
| Pro_OTU66 | Protist | 1.237 | Eukaryota(100);Amoebozoa(100);Lobosa(100);Tubulinea(100);Echinamoebida(100);Vermamoebidae(100);Hartmannella(100);Hartmannella_vermiformis(100); |
| Pro_OTU44 | Protist | 0.929 | Eukaryota(100);Amoebozoa(100);Lobosa(100);Tubulinea(100);Euamoebida(100);Hartmannellidae(100);Copromyxa(100);Copromyxa_protea(94); |
| Pro_OTU41 | Protist | 1.04 | Eukaryota(100);Rhizaria(100);Cercozoa(100);Filosa-Sarcomonadea(100);Glissomonadida(100);Sandonidae(100);unclassified;unclassified; |
| Pro_OTU1507 | Protist | 0.871 | Eukaryota(100);Rhizaria(100);Cercozoa(100);Filosa-Imbricatea(100);Euglyphida(100);Trinematidae(100);unclassified;unclassified; |
| Pro_OTU88 | Protist | 0.787 | Eukaryota(100);Rhizaria(100);Cercozoa(100);Endomyxa(100);Vampyrellida(100);Leptophryidae(100);Leptophryidae_X(100);Leptophryidae_X_sp.(100); |
| Pro_OTU57 | Protist | 0.733 | Eukaryota(100);Amoebozoa(100);Conosa(100);Variosea(100);Variosea_X(100);Filamoebidae(100);Filamoeba(100);Filamoeba_sp.(100); |
| Pro_OTU149 | Protist | 0.598 | Eukaryota(100);Rhizaria(100);Cercozoa(100);Filosa-Sarcomonadea(100);Glissomonadida(100);Sandonidae(100);unclassified;unclassified; |
| Pro_OTU72 | Protist | 0.525 | Eukaryota(100);Rhizaria(100);Cercozoa(100);Filosa-Sarcomonadea(97);Glissomonadida(97);Glissomonadida_X(97);Glissomonadida_XX(97);Glissomonadida_XX_sp.(97); |
| Pro_OTU71 | Protist | 0.466 | Eukaryota(100);Stramenopiles(100);Ochrophyta(100);Chrysophyceae(100);Chrysophyceae_X(100);Chrysophyceae_Clade-C(100);Spumella(100);Spumella_elongata(100); |
| Pro_OTU80 | Protist | 0.407 | Eukaryota(100);Amoebozoa(100);Lobosa(100);Tubulinea(100);Nolandida(99);Nolandellidae(99);Nolandellidae_X(99);Nolandellidae_X_sp.(99); |
| Pro_OTU148 | Protist | 0.475 | Eukaryota(100);Alveolata(100);Ciliophora(100);Colpodea(100);Colpodida(100);Colpodidae(100);Colpoda(100);unclassified; |
| Pro_OTU49 | Protist | 0.455 | Eukaryota(100);Rhizaria(100);Cercozoa(100);Filosa-Sarcomonadea(100);Glissomonadida(100);Allapsidae(100);Allapsidae_X(93);Allapsidae_X_sp.(93); |
| Pro_OTU53 | Protist | 0.466 | Eukaryota(100);Rhizaria(100);Cercozoa(100);Filosa-Sarcomonadea(100);Glissomonadida(100);Sandonidae(100);Sandonidae_X(98);Sandonidae_X_sp.(98); |
| Pro_OTU91 | Protist | 0.442 | Eukaryota(100);Stramenopiles(100);Ochrophyta(100);Chrysophyceae(100);Chrysophyceae_X(100);Chrysophyceae_Clade-C(100);Spumella(84);unclassified; |
| Pro_OTU135 | Protist | 0.377 | Eukaryota(100);Amoebozoa(100);Lobosa(100);Tubulinea(100);Nolandida(100);Nolandellidae(100);Nolandellidae_X(100);Nolandellidae_X_sp.(100); |
| Pro_OTU105 | Protist | 0.364 | Eukaryota(100);Rhizaria(100);Cercozoa(100);Filosa-Sarcomonadea(100);Cercomonadida(100);Cercomonadidae(100);Eocercomonas(100);Eocercomonas_sp.(100); |
| Pro_OTU54 | Protist | 0.39 | Eukaryota(100);Amoebozoa(100);Conosa(99);Variosea(99);Variosea_X(99);AND16-lineage(99);AND16-lineage_X(99);AND16-lineage_X_sp.(99); |
| Pro_OTU52 | Protist | 0.411 | Eukaryota(100);Rhizaria(100);Cercozoa(100);Filosa-Sarcomonadea(100);Cercomonadida(100);Cercomonadidae(100);Cercomonas(100);Cercomonas_sp.(100); |
| Pro_OTU111 | Protist | 0.414 | Eukaryota(100);Amoebozoa(100);Lobosa(100);Tubulinea(100);Echinamoebida(100);Vermamoebidae(100);unclassified;unclassified; |
| Pro_OTU60 | Protist | 0.375 | Eukaryota(100);Rhizaria(100);Cercozoa(100);Filosa-Sarcomonadea(100);Glissomonadida(100);Sandonidae(100);Neoheteromita(97);Neoheteromita_globosa(85); |
| Pro_OTU103 | Protist | 0.331 | Eukaryota(100);Amoebozoa(94);Conosa(94);Variosea(94);Variosea_X(94);Schizoplasmodiids(82);unclassified;unclassified; |
| Pro_OTU48 | Protist | 0.5 | Eukaryota(100);Alveolata(100);Ciliophora(100);Spirotrichea(100);Hypotrichia(100);Oxytrichidae(89);unclassified;unclassified; |
| Pro_OTU64 | Protist | 0.257 | Eukaryota(100);Alveolata(100);Ciliophora(100);Colpodea(100);Colpodida(100);Colpodidae(100);Colpoda(86);Colpoda_sp.(84); |
| Pro_OTU174 | Protist | 0.342 | Eukaryota(100);Rhizaria(100);Cercozoa(100);Filosa-Sarcomonadea(100);Glissomonadida(100);Allapsidae(100);Group-Te(100);Group_Te_sp.(100); |
| Pro_OTU75 | Protist | 0.324 | Eukaryota(100);Amoebozoa(100);Conosa(100);Variosea(100);Variosea_X(100);Acramoebidae(100);Acramoeba(100);Acramoeba_dendroida(100); |
| Pro_OTU122 | Protist | 0.276 | Eukaryota(100);Amoebozoa(100);Conosa(100);Variosea(100);Variosea_X(100);Mb5C-lineage(100);Mb5C-lineage_X(100);Mb5C-lineage_X_sp.(100); |
| Pro_OTU86 | Protist | 0.275 | Eukaryota(100);Amoebozoa(100);Conosa(100);Variosea(100);Variosea_X(100);Phalansteriidae(100);Phalansterium(100);Phalansterium_sp.(98); |
| Bac_OTU1 | Bacteria | 3.338 | Bacteria(100);Bacteroidetes(100);Bacteroidia(100);Chitinophagales(100);Chitinophagaceae(100);Chitinophagaceae_unclassified(100); |
| Bac_OTU3 | Bacteria | 2.214 | Bacteria(100);Gemmatimonadetes(100);Gemmatimonadetes(100);Gemmatimonadales(100);Gemmatimonadaceae(100);uncultured(96); |
| Bac_OTU7481 | Bacteria | 2.228 | Bacteria(100);Bacteroidetes(100);Bacteroidia(100);Chitinophagales(100);Chitinophagaceae(100);uncultured(91); |
| Bac_OTU5 | Bacteria | 1.955 | Bacteria(100);Bacteroidetes(100);Bacteroidia(100);Chitinophagales(100);Chitinophagaceae(100);Flavisolibacter(100); |
| Bac_OTU7 | Bacteria | 1.792 | Bacteria(100);Bacteroidetes(100);Bacteroidia(100);Chitinophagales(100);Chitinophagaceae(100);uncultured(83); |
| Bac_OTU4 | Bacteria | 1.573 | Bacteria(100);Gemmatimonadetes(100);Gemmatimonadetes(100);Gemmatimonadales(100);Gemmatimonadaceae(100);Gemmatimonadaceae_unclassified(100); |
| Bac_OTU2 | Bacteria | 1.521 | Bacteria(100);Proteobacteria(100);Gammaproteobacteria(100);Xanthomonadales(100);Rhodanobacteraceae(100);Rhodanobacter(96); |
| Bac_OTU8 | Bacteria | 1.258 | Bacteria(100);Gemmatimonadetes(100);Gemmatimonadetes(100);Gemmatimonadales(100);Gemmatimonadaceae(100);Gemmatimonas(96); |
| Bac_OTU12 | Bacteria | 1.03 | Bacteria(100);Acidobacteria(100);Acidobacteriia(100);Acidobacteriales(100);Koribacteraceae(98);Candidatus_Koribacter(98); |
| Bac_OTU6627 | Bacteria | 1.064 | Bacteria(100);Bacteroidetes(100);Bacteroidia(100);Chitinophagales(100);Chitinophagaceae(100);Chitinophagaceae_unclassified(100); |
| Bac_OTU26 | Bacteria | 1.064 | Bacteria(100);Proteobacteria(100);Gammaproteobacteria(100);Betaproteobacteriales(100);SC-I-84(100);SC-I-84_ge(100); |
| Bac_OTU6 | Bacteria | 1.033 | Bacteria(100);Bacteroidetes(100);Bacteroidia(100);Chitinophagales(100);Chitinophagaceae(100);uncultured(87); |
| Bac_OTU9 | Bacteria | 0.862 | Bacteria(100);Acidobacteria(100);Blastocatellia_(Subgroup_4)(100);Blastocatellales(100);Blastocatellaceae(100);uncultured(83); |
| Bac_OTU11 | Bacteria | 0.811 | Bacteria(100);Verrucomicrobia(100);Verrucomicrobiae(100);Chthoniobacterales(100);Chthoniobacteraceae(100);Chthoniobacteraceae_unclassified(100); |
| Bac_OTU24 | Bacteria | 0.854 | Bacteria(100);Gemmatimonadetes(100);Gemmatimonadetes(100);Gemmatimonadales(100);Gemmatimonadaceae(100);Gemmatimonas(98); |
| Bac_OTU14 | Bacteria | 0.865 | Bacteria(100);Nitrospirae(100);Nitrospira(100);Nitrospirales(100);Nitrospiraceae(100);Nitrospira(100); |
| Bac_OTU10 | Bacteria | 0.811 | Bacteria(100);Bacteroidetes(100);Bacteroidia(100);Chitinophagales(100);Chitinophagaceae(100);Flavitalea(100); |
| Bac_OTU15 | Bacteria | 0.773 | Bacteria(100);Acidobacteria(100);Acidobacteriia(100);Acidobacteriales(100);uncultured(99);uncultured_ge(99); |
| Bac_OTU29 | Bacteria | 0.723 | Bacteria(100);Acidobacteria(100);Acidobacteriia(100);Acidobacteriales(100);Acidobacteriaceae_(Subgroup_1)(100);Acidipila(97); |
| Bac_OTU158 | Bacteria | 0.734 | Bacteria(100);Bacteroidetes(100);Bacteroidia(100);Chitinophagales(100);Chitinophagaceae(100);uncultured(90); |
| Bac_OTU17 | Bacteria | 0.692 | Bacteria(100);Bacteroidetes(100);Bacteroidia(100);Chitinophagales(100);Chitinophagaceae(100);Terrimonas(81); |
| Bac_OTU22 | Bacteria | 0.611 | Bacteria(100);Bacteroidetes(100);Bacteroidia(100);Chitinophagales(100);Saprospiraceae(100);uncultured(100); |
| Bac_OTU25 | Bacteria | 0.613 | Bacteria(100);Acidobacteria(100);Acidobacteriia(100);Acidobacteriales(100);Acidobacteriaceae_(Subgroup_1)(100);Terracidiphilus(86); |
| Bac_OTU23 | Bacteria | 0.603 | Bacteria(100);Acidobacteria(100);Acidobacteriia(98);Acidobacteriales(98);Acidobacteriales_unclassified(98);Acidobacteriales_unclassified(98); |
| Bac_OTU21 | Bacteria | 0.584 | Bacteria(100);Bacteroidetes(100);Bacteroidia(100);Chitinophagales(100);Chitinophagaceae(100);UTBCD1(99); |
| Bac_OTU70 | Bacteria | 0.559 | Bacteria(100);Proteobacteria(100);Deltaproteobacteria(100);Deltaproteobacteria_unclassified(100);Deltaproteobacteria_unclassified(100);Deltaproteobacteria_unclassified(100); |
| Bac_OTU5377 | Bacteria | 0.506 | Bacteria(100);Chloroflexi(100);Ktedonobacteria(100);Ktedonobacterales(100);JG30-KF-AS9(100);JG30-KF-AS9_ge(100); |
| Bac_OTU20 | Bacteria | 0.568 | Bacteria(100);Bacteroidetes(100);Bacteroidia(100);Sphingobacteriales(100);Sphingobacteriaceae(100);Sphingobacteriaceae_unclassified(100); |
| Bac_OTU104 | Bacteria | 0.503 | Bacteria(100);Bacteroidetes(100);Bacteroidia(100);Chitinophagales(100);Chitinophagaceae(100);Chitinophagaceae_unclassified(100); |
| Bac_OTU6804 | Bacteria | 0.477 | Bacteria(100);Gemmatimonadetes(100);Gemmatimonadetes(100);Gemmatimonadales(100);Gemmatimonadaceae(100);Gemmatimonas(100); |
| COG1228 | Q gene | 0.167 | amidohydrolase |
| COG0500 | Q gene | 0.114 | methyltransferase |
| COG0179 | Q gene | 0.114 | Fumarylacetoacetate hydrolase |
| COG1233 | Q gene | 0.087 | phytoene |
| COG2132 | Q gene | 0.084 | Multicopper oxidase |
| COG3653 | Q gene | 0.079 | Deacylase |
| COG0412 | Q gene | 0.075 | dienelactone hydrolase |
| COG1020 | Q gene | 0.063 | non-ribosomal peptide synthetase |
| COG1127 | Q gene | 0.058 | (ABC) transporter |
| NOG00295 | Q gene | 0.056 | Amp-dependent synthetase and ligase |
| COG0767 | Q gene | 0.055 | (ABC) transporter |
| COG2124 | Q gene | 0.054 | Cytochrome p450 |
| COG3396 | Q gene | 0.051 | Phenylacetate-CoA oxygenase, subunit |
| COG3321 | Q gene | 0.044 | synthase |
| COG1541 | Q gene | 0.044 | Phenylacetate-CoA ligase |
| COG3508 | Q gene | 0.044 | Homogentisate 1,2-dioxygenase |
| COG2050 | Q gene | 0.038 | thioesterase Superfamily protein |
| COG4664 | Q gene | 0.034 | trap dicarboxylate transporter dctm subunit |
| COG1335 | Q gene | 0.033 | isochorismatase |
| COG3485 | Q gene | 0.031 | protocatechuate 3,4-dioxygenase |
| NOG01415 | Q gene | 0.029 | amino acid adenylation |
| COG4663 | Q gene | 0.027 | extracellular solute-binding protein, family 7 |
| COG2175 | Q gene | 0.025 | dioxygenase |
| COG3127 | Q gene | 0.023 | ABC transporter (permease) |
| COG4181 | Q gene | 0.022 | (ABC) transporter |
| COG2931 | Q gene | 0.019 | Hemolysin-type calcium-binding |
| NOG03738 | Q gene | 0.019 | phenylacetic acid degradation protein |
| COG2368 | Q gene | 0.019 | 4-hydroxyphenylacetate |
| NOG02007 | Q gene | 0.016 | Protocatechuate 4,5-dioxygenase |
| COG2313 | Q gene | 0.015 | Catalyzes the hydrolysis of pseudouridine 5'-phosphate (PsiMP) to ribose 5-phosphate and uracil (By similarity) |

Because only 3 replicates of meta-genomic sequencing data was identically matched with the protistan and bacterial data, we used the 30 samples in total (2 symptoms * 5 time points * 3 replicates) for the analyses. RA = average relative abundance across the 30 samples.

**Table S6. Topological properties of networks between the abundant phagotrophic protistan OTUs, bacterial OTUs and metabolism Q genes for healthy and diseased plants across plant growth.**

|  | **Healthy plants** | **Diseased plants** |
| --- | --- | --- |
| **Number of total nodes** | 55 | 41 |
| Number of phagotrophic protistan OTUs | 8 | 6 |
| Number of bacterial OTUs | 29 | 27 |
| Number of metabolism Q genes | 18 | 8 |
| **Positive correlations (links)** | 71 | 50 |
| **Negative correlations (links)** | 19 | 9 |
| **Edge density** | 0.061 | 0.072 |
| **Mean distance** | 2.139 | 1.670 |
| **Clustering coefficient** | 0.695 | 0.713 |
| **Modularity** | 0.698 | 0.713 |


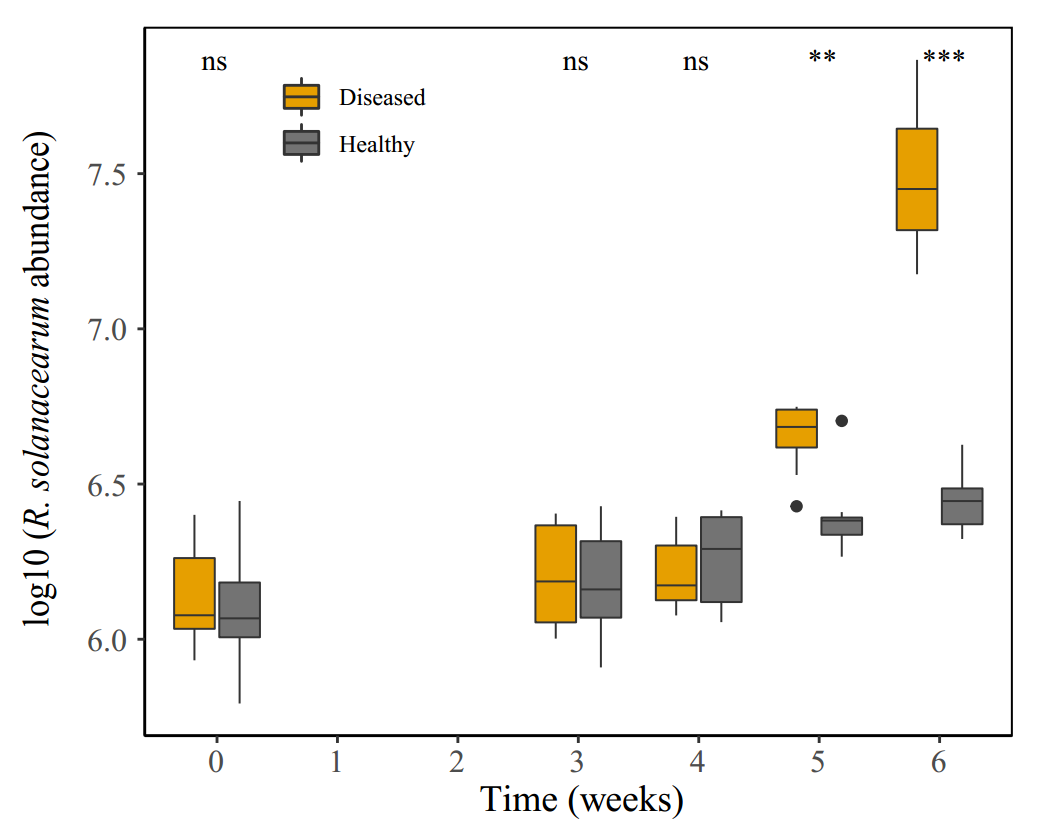


**Fig. S1 Abundance of *R. solanacearum* pathogen in diseased and healthy plants across plant growth.**

“ns” means not significant, **means *P* < 0.01 and ***means *P* < 0.001 under student’s t-test (n = 8).


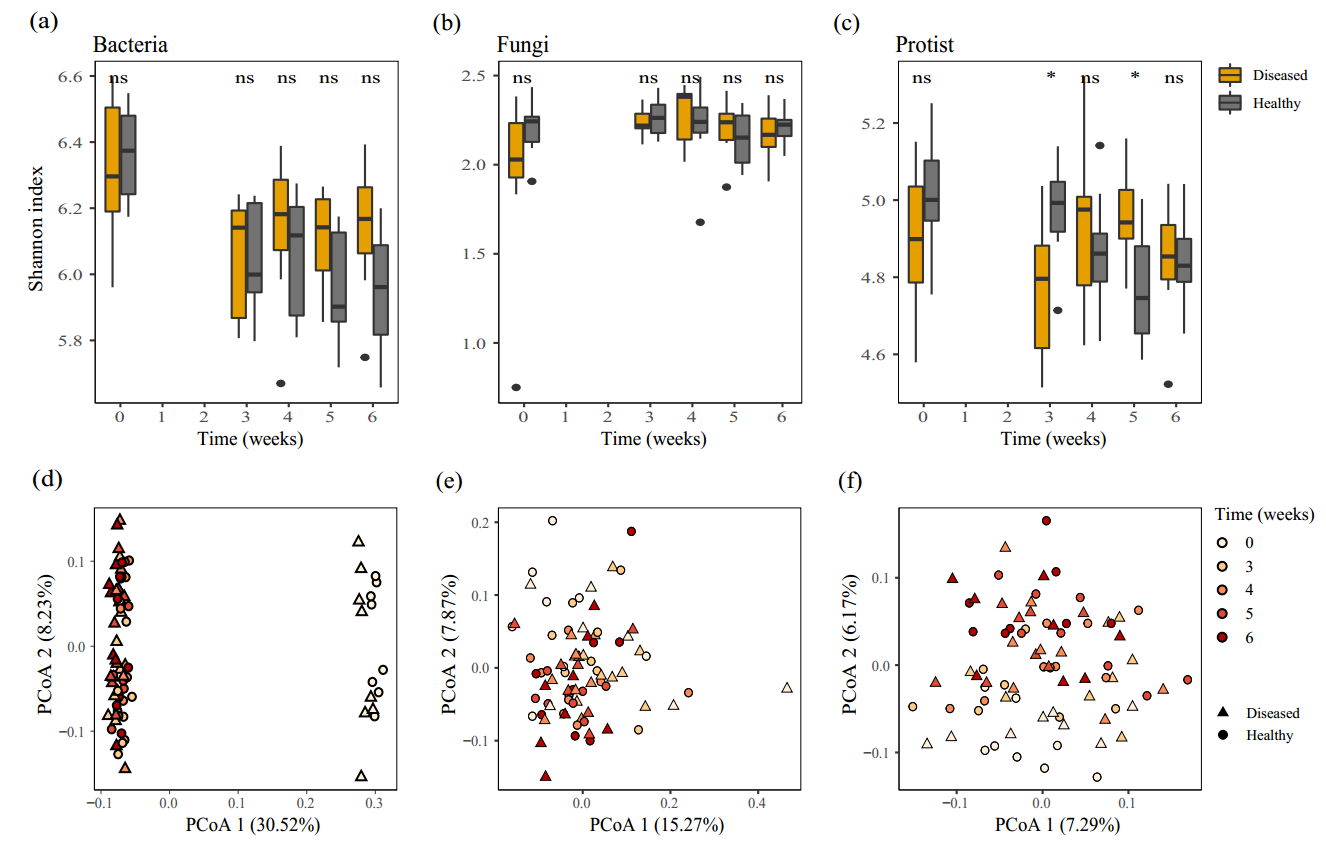


**Fig. S2 Shannon diversity of bacteria (a), fungi (b) and protists (c) in diseased and healthy plants across plant growth, and overall community structures of bacteria (d), fungi (e) and protists (f) in diseased and healthy plants across plant growth.**

“ns” means not significant, *means *P* < 0.05 under student’s t-test (n = 8).


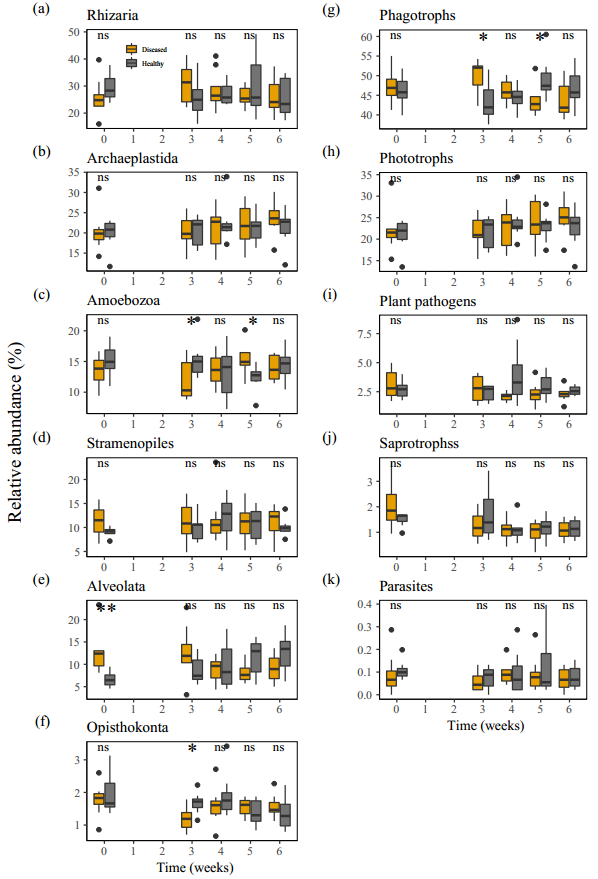


**Fig. S3 Relative abundances of the most abundant (average relative abundance over 1% across all samples) taxonomic (a, b, c, d, e and f) and functional (g, h, i, j and k) protistan groups in diseased and healthy plants across plant growth.**

“ns” means not significant, *means *P* < 0.05 and **means *P* < 0.01 under student’s t-test (n = 8).


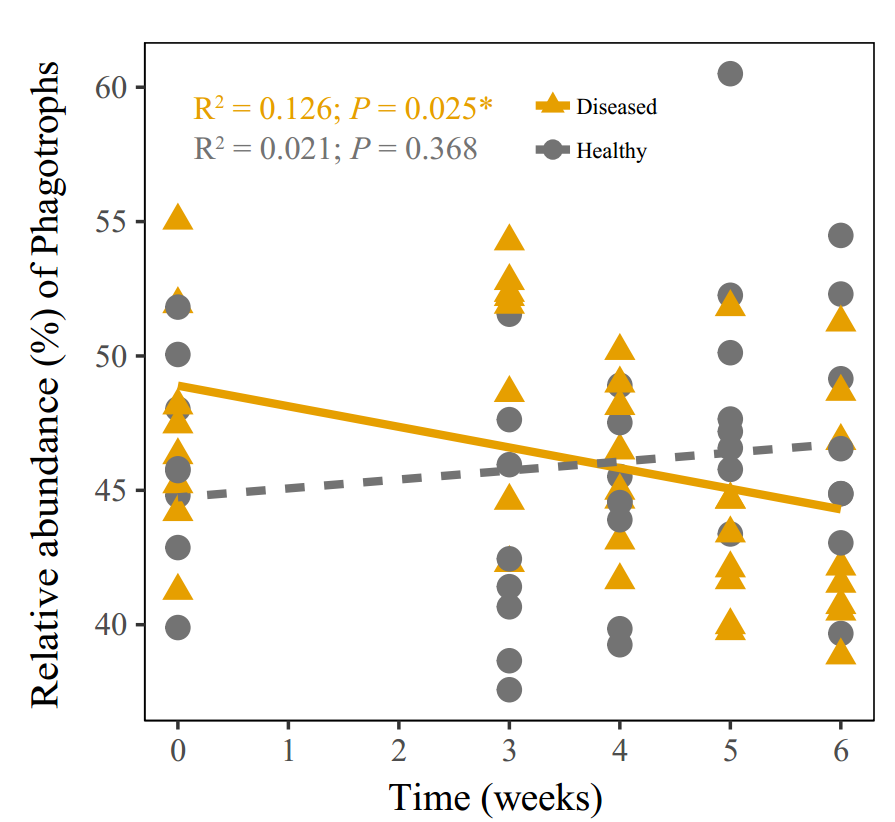


**Fig. S4 Correlation between the relative abundance of phagotrophic protists and plant growth time in diseased and healthy plants.**

The solid line shows a significant (*P* < 0.05) correlation and the dashed line shows the non-significant (*P* > 0.05) correlation.


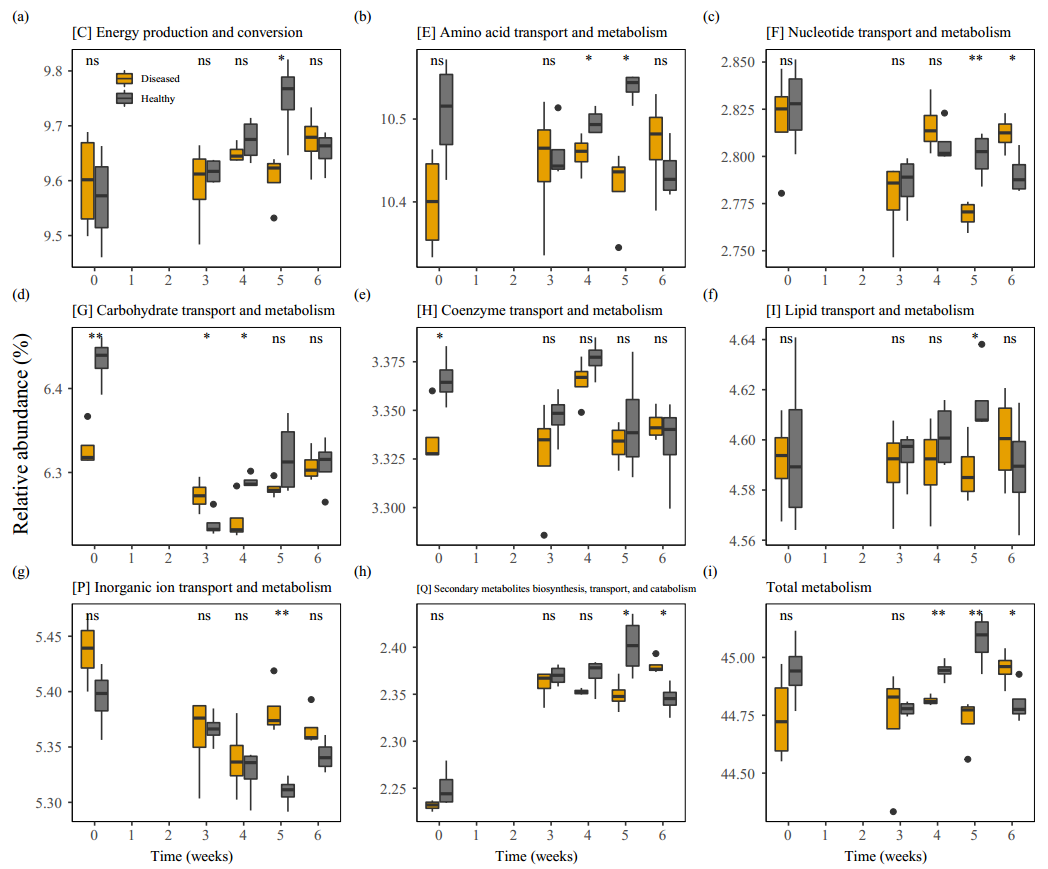


**Fig. S5 Relative abundances of the eight metabolism genes and total metabolism genes in diseased and healthy plants across plant growth.**

[C] Energy production and conversion (a), [E] Amino acid transport and metabolism (b), [F] Nucleotide transport and metabolism (c), [G] Carbohydrate transport and metabolism (d), [H] Coenzyme transport and metabolism (e), [I] Lipid transport and metabolism (f), [P] Inorganic ion transport and metabolism (g), [Q] Secondary metabolite biosynthesis, transport, and catabolism (h) and total metabolism genes (i). “ns” means not significant, *means *P* < 0.05 and **means *P* < 0.01 under student’s t-test (n = 4).


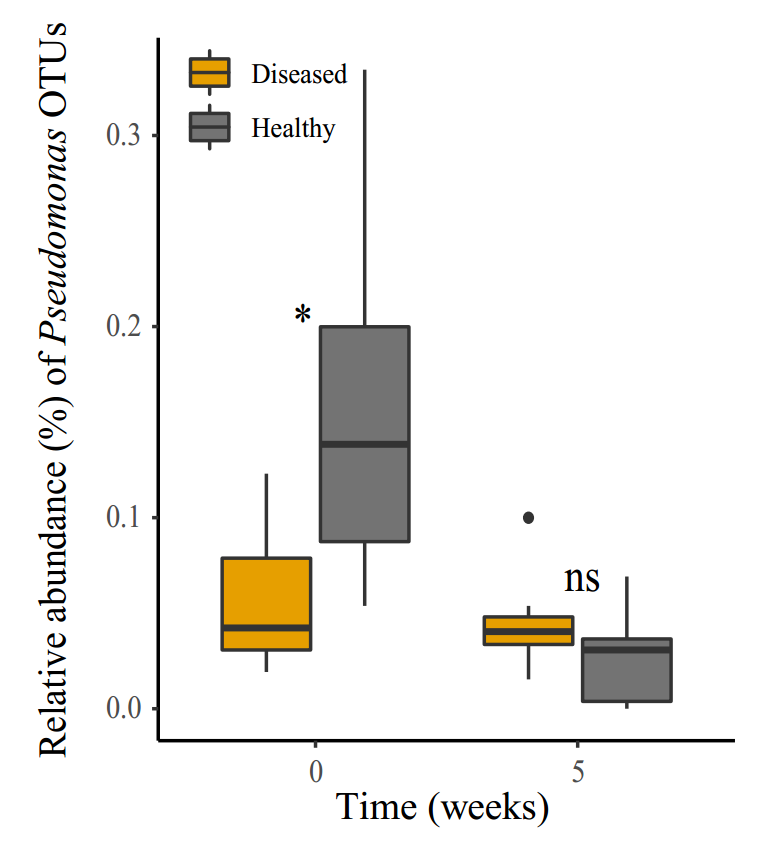


**Fig. S6 Relative abundance of *Pseudomonas* OTUs in diseased and healthy plants at week 0 and week 5.**

The relative abundance of *Pseudomonas* OTUs combines the four *Pseudomonas* OTUs from the bacterial OTU table. “ns” means not significant, *means *P* < 0.05 under student’s t-test (n = 8).
